# The Effect of Risk Factors on the Levels of Chemical Elements in the Tibial Plateau of Patients with Osteoarthritis following Knee Surgery

**DOI:** 10.1155/2015/650282

**Published:** 2015-10-25

**Authors:** Natalia Lanocha-Arendarczyk, Danuta Izabela Kosik-Bogacka, Adam Prokopowicz, Elzbieta Kalisinska, Sebastian Sokolowski, Maciej Karaczun, Pawel Zietek, Joanna Podlasińska, Bogumila Pilarczyk, Agnieszka Tomza-Marciniak, Irena Baranowska-Bosiacka, Izabela Gutowska, Krzysztof Safranow, Dariusz Chlubek

**Affiliations:** ^1^Department of Biology and Medical Parasitology, Pomeranian Medical University, Powstancow Wielkopolskich 72, 70-111 Szczecin, Poland; ^2^Department of Chemical Hazards and Genetic Toxicology, Institute of Occupational Medicine and Environmental Health, Koscielna 13, 71-200 Sosnowiec, Poland; ^3^Chair and Clinic of Orthopaedics and Traumatology, Pomeranian Medical University, Unii Lubelskiej 1, 71-252 Szczecin, Poland; ^4^Department of Ecology, Environmental Management and Protection, Slowackiego 17, 71-434 Szczecin, Poland; ^5^Department of Animal Reproduction Biotechnology and Environmental Hygiene, West Pomeranian University of Technology, Doktora Judyma 6, 71-466 Szczecin, Poland; ^6^Department of Biochemistry and Medical Chemistry, Pomeranian Medical University, Powstancow Wielkopolskich 72, 70-111 Szczecin, Poland; ^7^Department of Biochemistry and Human Nutrition, Pomeranian Medical University, Broniewskiego 24, 71-460 Szczecin, Poland

## Abstract

The aim of this study was to evaluate the aforementioned chemical elements in tibial plateau samples obtained during knee arthroplasty. The gender-specific analysis of chemical element levels in the bone samples revealed that there were statistically significant differences in the concentration of Pb and Se/Pb ratio. The contents of elements in the tibial plateau in the patients with osteoarthritis (OA) can be arranged in the following descending order: F^−^ > K > Zn > Fe > Sr > Pb > Mn > Se > Cd > THg. We observed statistical significant effects of environmental factors including smoking, seafood diet, and geographical distribution on the levels of the elements in tibial bone. Significant positive correlation coefficients were found for the relationships K-Cd, Zn-Sr, Zn-F^−^, THg-Pb, Pb-Cd, Se-Se/Pb, Se-Se/Cd, Se/Pb-Se/Cd, Pb-Cd/Ca, Cd-Cd/Ca, and F^−^-F^−^/Ca·1000. Significant negative correlations were found for the relationships THg-Se/Pb, Pb-Se/Pb, Cd-Se/Pb, K-Se/Cd, Pb-Se/Cd, Cd-Se/Cd, THg-Se/THg, Pb-Se/THg, Se-Pb/Cd, Zn-Cd/Ca, and Se/Cd-Cd/Ca. The results reported here may provide a basis for establishing reference values for the tibial plateau in patients with OA who had undergone knee replacement surgery. The concentrations of elements in the bone with OA were determined by age, presence of implants, smoking, fish and seafood diet, and sport activity.

## 1. Introduction

Osteoarthritis (OA), the most common form of arthritis, is a serious disease affecting the joints due to an imbalance between the processes of degeneration and regeneration of cartilage structures [1, 2]. Age-related and incurable OA is manifested by gradual degenerative arthritis, leading to premature motor disability.

The pathogenesis of osteoarthritic changes in the joints is complex, with metalloproteinases playing the central role in the degeneration of cartilage, although genetic and environmental factors such as age, gender, body mass, and sport activity are also mentioned [3]. The presence of OA in the knee is highly age-related but gender-specific differences are also evident. In persons younger than 50 years of age, the prevalence of this disease is higher in men [2]. OA is predicted to reach 30% of the population above 60 years of age by the year 2030 [4]. The symptoms of OA are stronger in women over 50 and in the postmenopausal period. Clinical symptoms include joint pain, tenderness, restriction of movement, loss of articular cartilage, sclerosis, and increased density of subchondral bone [5].

Many metals that are present in organisms accumulate in the bones and may be released due to various pathological conditions [6]. There is little research focusing on the element composition of bone or on correlations between metals in joint cartilage and bone tissues in patients with osteoarticular degeneration, including OA [7–10]. These few analyses usually examine the cancellous bone portions of patients following hip arthroplasty or the ribs of healthy people [8, 11]. The choice of the cancellous bone is related to its high sensitivity to hormones and other biological factors, and the onset of OA can cause cartilage loss and subchondral sclerosis [12]. However, the tibial plateau, which consists mainly of cortical bone, can also be an original source of material for biomonitoring research, especially due to its features that provide structural integrity-strength, density, and compactness.

Cadmium (Cd), lead (Pb), and mercury (THg) often cause acute and chronic environmental contamination and inflict damage primarily to the kidney, liver, brain, and bone. In one of the most drastic cases, in the 1950s in Japan, Cd poisoning resulted in the Itai-itai disease, a mixed pattern of bone diseases and damage to the kidney [10]. Over a lifetime, 90% of Pb will accumulate in the bones. Little is known about the effects of total THg on the human skeleton and concentrations in bone samples of the knee joint and/or hip joint. Moreover, interactions between the aforementioned metals can also affect their toxicity.

Prolonged exposure to large amounts of fluoride (F) in inhaled air or in the diet results in a deposition of fluoride ions (F^−^) in bone tissue, mainly in cancellous bone. Fluoride reduces Ca absorption, leading to impaired mineralization of newly formed osteoid [13]. In contrast, iron (Fe), zinc (Zn), selenium (Se), and manganese (Mn) are considered by WHO to be essential elements for the correct functioning of living organisms but may be toxic at high levels of exposure [14]. The deficiencies of some of these elements can increase the accumulation and toxicity of various toxic metals. Fe and Zn deficiencies, for instance, increase the susceptibility to Cd and Pb toxicity [15]. Although strontium (Sr) stimulates bone formation and inhibits osteoclastic activity, it has not been proven to be essential. In hydroxyapatite crystal, Sr is antagonist to Ca [12, 16, 17]. For some years it has also been suggested that potassium (K) exerts a beneficial effect on the skeleton through anions provided by potassium salts and the anion-independent effect of K on Ca excretion and bone metabolism [18].

The aim of this present study was to evaluate the aforementioned 10 chemical elements in tibial plateau samples obtained during knee arthroplasty from patients from northern and western Poland. Furthermore, the aim was to examine synergistic and/or antagonistic relationships between chemical elements in the bone samples and environmental exposure factors, as well as examining the effects of environmental factors including smoking, diet, occupational exposure, sport activity, and supplementation, on the levels of the elements in tibial bone diagnosed with OA.

## 2. Materials and Methods

For this study 33 tibial plateau samples were examined. The specimens were obtained from women (*n* = 22) aged from 54 to 83 years (mean age 67.0) and men (*n* = 11) aged from 48 to 79 years (mean age 64.5) following knee joint arthroplasty. These patients had been hospitalized at orthopedic clinics in the West Pomeranian (WV) in Szczecin (WVS) and Koszalin (WVK) and Lubuskie (LV) voivodships in 2013-2014. The tibial plateau samples had been acquired intraoperatively from patients who had undergone knee joint arthroplasty due either to osteoarthritis (OA, *n* = 28) or knee injury (KI, *n* = 5). Patients with knee injuries were subject to knee replacement surgery due to mechanical trauma (accidents); the group included males (*n* = 3; mean age: 53.0) and females (*n* = 2; mean age: 59.5). The analysis excluded patients who had been treated with fluoride-containing preparations. All the patients were interviewed using a questionnaire to collect data on demographics, health status, occupational exposure to potentially toxic elements, smoking, diet, sport activity and supplementation. Among the patients, 18 persons had been cigarette smokers for at least 20 years, while 15 persons were non-smokers. The use of the bone tissue in the research was approved by the Bioethics Committee of Pomeranian Medical University in Szczecin (KB-0012/78/13).

Prior to analysis, the tissue samples had been stored at a temperature below −20°C. The sampled bones were cleaned of adhering soft tissue and marrow, and degreased with acetone (Chempur, Poland) for 3 h. Bone samples were dried to a constant weight at 55°C (for the determination of THg) and 105°C (for the determination of Se, F^−^, Fe, Mn, Zn, Sr, Cd, Pb, Ni, K) for 3 weeks in a drying oven with forced air circulation (Binder GmbH, Germany) so that water content could be determined (gravimetric method), and then crushed in an agate mortar. Selenium concentrations in the bone samples were determined by spectrofluorimetric method (RF 5001 PC Shimadzu spectrophotofluorometer) according to Pilarczyk et al. [19]. Determination of THg concentrations was performed by atomic absorption spectroscopy (AAS) using an AMA 254 mercury analyzer. More details on the analytical procedures are available in our previous research [10]. Fluoride concentrations were determined by potentiometric method using an Orion ion-selective F electrode, as described by Palczewska-Komsa et al. [20]. To determine the levels of the other elements, a separated sample (0.3 g) of dried bone tissue was placed in a PFA dish. 1.5 mL of concentrated nitric acid 69.0–70.0% (Avantor Performance Materials, J.T. Baker Analyzed Instra Reagent, USA) was added to the samples which were then digested in on a hot plate at 120°C for 16 hours. After cooling, 1.5 mL of 30% hydrogen peroxide (Baker Analyzed) was added, and the digestion continued for another 24 hours. The resulting sample was diluted with deionized water (milliQ) to 15 mL and analyzed for Pb, Cd, Sr, K, Zn, Mn, and Fe concentrations. The reference material and blank samples were prepared by the same digestion procedure. Lead, Cd, and Mn levels were determined by atomic absorption spectrometry with electrothermal atomization in a graphite furnace (GFAAS) using a PerkinElmer 4100ZL spectrometer (Perkin-Elmer Bodenseewerk, Überlingen, Germany) equipped with an autosampler and Zeeman background correction. Temperature and time programs for the graphite furnace were optimized for each element. Zn and Fe were determined by flame atomic absorption spectrometry (FAAS) in an air-acetylene flame using a UNICAM 939 Solaar atomic absorption spectrometer equipped with a deuterium background correction. Fe was determined using standard solutions prepared from the matrix of the sample. K and Sr were determined by flame atomic emission spectrometry (FAES) using a UNICAM 939 Solaar spectrometer and an air-acetylene flame to measure K or a nitrous oxide-acetylene flame to determine Sr. In both assays, ionization buffers were used: a 0.2% solution of sodium (NaCl) and a 0.2% solution of potassium (KCl), respectively. To validate the analytical techniques, reference samples NIST-SRM 1486 (Bone meal) and Fish Tissue IAEA-407 were used. The analytical values were within the range of certified values. Recoveries consistently remained in the range 88–97%. The concentrations of elements were expressed as mg/kg on dry mass basis (dm).

Statistical analysis was carried using the Statystyka PL program. The distribution normality was examined using a Shapiro-Wilk test. Due to the nonparametric distribution of results, intergroup comparisons were performed using the Mann-Whitney  *U* test. The correlations between the analyzed variables were calculated with Spearman's rank correlation coefficient (*r*_*s*_). The significance level was *p* < 0.05.

## 3. Results

The gender-specific analysis of chemical element levels in the bone samples revealed that there were statistically significant differences in the concentration of Pb and in the Se/Pb ratio (Tables 1 and 2). Lead concentrations were 1.74 times higher in the men than in the women. Likewise, Se/Pb ratio was higher in the men than in the women (Table 2). There were no statistically significant differences in the group of potential essential elements between the genders.

A comparison of chemical element levels between the two groups of patients with OA and KI showed statistically significant differences only in the F^−^ concentration in the inorganic portion of bone: F^−^/Ca·1000 (*U* = 29; *p* < 0.01) and in age (*U* = 11.0, *p* < 0.001). Differences close to statistical significance (*p* = 0.06) were observed in F^−^ level and Se/THg ratio, as well as in relation to the number of dental amalgams in the OA group. In the tibial plateau, the concentrations of Sr, F^−^, and Cd were higher in the OA patients than in the KI, while Zn and Mn levels were, respectively, lower, but these differences were not statistically significant.

A comparison of element levels in the bone between patients from northwestern Poland (WVS and WSK) and western Poland (LV) showed statistically significant differences only in Se levels, between LV and WVS (*U* = 6.0; *p* < 0.01), and between WVS and WVK (*U* = 3.0; *p* < 0.01). The highest Se concentrations were observed in the tibial plateau samples of the patients from WVS (0.047 mg/kg dm) and then from LV (0.037 mg/kg dm) and the lowest were found in those from WVK (0.024 mg/kg dm).

Between the patients occupationally exposed (OE) or unexposed (UE) to potentially toxic elements (Pb, THg, and Cd) no statistically significant differences were observed in bone tissue sample levels. However, the concentrations of Pb, THg in the OE patients who had undergone knee surgery were, respectively, more than 1.4 and 1.7 times higher than in the UE patients, at 2.40 versus 1.74, and 0.0067 versus 0.0040 mg/kg dm, respectively.

Analysis of element levels in the bone samples in relation to smoking (elements contained in cigarettes were taken into account) showed statistically significant differences in the concentrations of Pb and Cd in the smokers, with significantly higher ratios of Se/Pb, Se/Cd, and Se/THg, in the nonsmoking patients (Table 3). The widest differences were observed in Pb levels between the groups of smokers and nonsmokers.

The patients with dental amalgam fillings (DA) who had been exposed mainly to THg, Cd, and Zn contained in amalgams showed no statistically significant differences in element levels in bone tissue compared to the patients without amalgam fillings (NDA), despite higher THg concentrations in the patients with DA (over 1.5 times higher) than the NDA patients (0.0061 versus 0.0039 mg/kg dm). Furthermore, in terms of age and the number of dental amalgams, there were significant differences between the DA and NDA groups (*U* = 35.0, *p* < 0.01 and *U* = 62.5, *p* < 0.01, resp.).

The comparison of element levels in the tibial plateau between those who consumed fish and seafood (FD) and those who did not (NFD) showed statistically confirmed differences in Se, THg concentrations, and Se/THg ratio (*U* test: M-W = 71.5, *p* < 0.04; 60.0, *p* < 0.01; 73.0, *p* < 0.04, resp.). The lowest concentrations of Se and THg were found in the NFD group (0.036 and 0.004 mg/kg dm), compared to the FD group (0.042 and 0.006 mg/kg dm). In the tibial plateau samples from the FD group patients there was a lower Se/THg ratio compared to the NFD group participants (10.84 and 21.57, resp.).

A comparison of the mean levels of chemical elements showed no statistically significant differences between the participants who preferred dairy products to those who did not consume milk and cheese.

Moreover, there were significant differences in the concentrations of Se between the patients who used dietary supplements (WS) and those who did not (NS) (*U* = 57.5, *p* < 0.02). We also observed a difference in the concentration of Mn, which was close to statistical significance (*U* = 67.0, *p* = 0.06). Se and Mn concentrations for WS (0.047 and 0.38 mg/kg dm) were 1.4 and 1.3 times higher than for NS (0.034 and 0.29 mg/kg dm, resp.). Differences in chemical element contents in the bone samples between the patients with and without implants only proved to be statistically significant with regard to Fe level (*U* = 30.0, *p* < 0.01), at 106.13 and 45.09 mg/kg dm, respectively.

The greatest positive correlations were found between the concentrations of Pb and/or Cd/Ca, and the number of cigarettes smoked (Table 4). Correlation analysis showed an inverse correlation between Se/Pb ratio in the cigarette smokers, Se/THg ratio, and a fish and seafood diet. Negative correlation close to significance between Se and sport activity was observed in the patients with OA.

Significant positive correlation coefficients were found for the relationships K-Cd, Zn-Sr, Zn-F^−^, THg-Pb, Pb-Cd, Se-Se/Pb, Se-Se/Cd, Se/Pb-Se/Cd, Pb-Cd/Ca, Cd-Cd/Ca, and F^−^-F^−^/Ca·1000. Significant negative correlations were found for the relationship THg-Se/Pb, Pb-Se/Pb, Cd-Se/Pb, K-Se/Cd, Pb-Se/Cd, Cd-Se/Cd, THg-Se/THg, Pb-Se/THg, Se-Pb/Cd, Zn-Cd/Ca, and Se/Cd-Cd/Ca.

## 4. Discussion

Some publications indicate that the levels of potentially toxic elements in bone depend on the gender of participants. In this study, the Pb concentration and Se/Pb ratio were higher in the men than in the women (Table 1). Similarly, in a previous analysis we observed that Pb concentration in the cartilage with cortical bone was about 28% higher in men than in women [10]. Also S. Zaichick and V. Zaichick [21] and Kubaszewski et al. [22] report higher Pb concentrations of males compared to females in the ribs and femoral head, respectively.

In the case of certain medical conditions including OA, osteoporosis, bone cancers, Ewing's sarcoma, and osteomyelitis, there are reports of both typical as well as reduced or elevated concentrations of various trace elements [8, 23–25]. In this study, element levels in the tibial plateau in the patients with OA can be arranged in the following descending order: F^−^ > K > Zn > Fe > Sr > Pb > Mn > Se > Cd > THg. A slightly different order in OA patients is presented by Lanocha et al. Fe > Zn > Sr > Se > Mn > Pb; Se and Pb concentrations in bone without osteoarticular degeneration are over 4 times higher than in bone with osteoporosis. In this research, the concentrations of Zn, Pb, Cd, and THg in cancellous bone of the patients with osteoporosis were 1.3, 3.7, 1.9, 1.5, and 2 times higher than in our previous research [24].

Osteoporosis and OA are both common conditions with a high age-related prevalence, and a large variety of intrinsic and extrinsic factors are involved in both diseases. Thomsen et al. [26] found that patients with OA have significantly higher Zn levels in serum and lower Zn levels in urine than osteoporotic patients, whereas Zn level in bone does not differ, which is consistent with the levels of Zn in the bone of the patients with OA and KI evaluated in this study. Zn concentrations in the bone samples were the same in both groups of patients with OA and KI, probably because Zn is firmly bound to bone and is not easily released in the case of a negative Zn balance. In our study the concentrations of Zn and Pb in the tibial plateau were higher than in the femur head in patients with OA [22].

Long-term exposure to Cd may result in skeletal disorders, that is, osteoporosis or osteomalacia with an increased number of bone fractures [8]. Wiechuła et al. [27] report that Pb and Cd concentrations in the femoral head of patients with coxarthrosis is equal to about 3 mg/kg and 0.07 mg/kg, respectively, and is higher than in the tibial plateau of patients with OA evaluated in this study. However, the Cd concentration in cortical bone from the femur head with coxarthrosis [28] is similar to the concentration of Cd in the tibial plateau of patients with OA. Much higher Cd levels are found in patients with degenerative changes of the hip joint from the Upper Silesian Region [8].

In this study, we observed a weak correlation between F^−^/Ca·1000 and the age of the patients. Bohatyrewicz [13] proved that F^−^/Ca·1000 is correlated with age and shows higher values in patients who had trochanteric fracture in older age. This could be due to the artificial fluoridation of drinking water, which ceased in Poland in the 1990s. The permissible F^−^ concentration in water in Poland is 1.5 mg·L^−1^, while in tap water from the area of northwestern Poland, F^−^ concentration does not exceed 0.3 mg·L^−1^ [29]. Currently, in accordance with applicable law, measurements of air fluoride concentrations are not carried out in Poland or in other countries of the European Union.

The concentrations of elements are varied and depend on the geographical location. Se concentrations in human tissues obtained from adults in Japan, USA, and Canada are much higher than those in Poland, as they depend on the geographical location which determines Se content in the diet [30]. A large part of Europe, including some regions of Poland, is considered to be deficient in Se [19]. In our research, we observed a significant difference in the Se levels of Se patients coming from different parts of northern and western Poland. Low levels of Se (<0.05 mg/kg dm) contained in bone samples are probably an effect of its deficiency in the diet and depend on the region of the country. In Poland, the levels of Se in plasma depend on the geographical location; in the central, northern and northwestern parts they are estimated at 78.0, 72.3, and 54.8 *μ*g/L, respectively [31].

In this study, the concentrations of Pb and THg in the OE patients following knee surgery were over 1.4 and 1.7 times higher than in the UE participants. In 2005, the emissions of Pb, Cd, and THg in Poland reached 526 t, 46 t, and 20.1 t, with the highest Pb, Cd, and THg emissions reported in the following voivodships: Lower Silesian and Silesian (167 t; 8 t, and 3.5 t) while the lowest in Lubuskie (3.7 t, 0.9 t, and 0.2 t) [15]. The largest concentrations of Cd were observed in patients from Upper Silesia and Cracow. They were over 20 and 1.3 times higher than in the occupationally exposed participants examined in this study [6, 8]. Kwapuliński et al. [32] state that cortical bone from the femur head of patients from the Upper-Silesian Industrial District contained the highest concentrations of THg ever reported in Poland—20 times higher than the THg levels found in the OE patients examined in this study.

Compared to data on Western Pomerania from our 2012 study, Pb and Cd levels in the cartilage and compact bone of the femoral head were lower by nearly 5 and 2 times than the levels observed in the OE patients [10]. Even higher Cd concentrations in bone were found in people living in highly industrialized Taiwan [33]. In patients living in various Asian countries (including South Korea and Taiwan) who had not been occupationally exposed to Pb and THg, there were much higher levels of Pb and THg than in the participants of our study [34].

Cigarette smoking is a major exposure route for Cd and Pb in the general population. Little is known about the effect of smoking on the pathogenesis and progression of symptomatic osteoarthritis of the knee, one of the leading causes of disability in elderly people. Amin et al. [35] indicate that men with symptomatic knee osteoarthritis who smoke have an increased risk of articular cartilage loss and more severe knee pain than men who do not smoke. In our study we found that Pb and Cd concentrations were higher in the group of smokers than in nonsmokers. In scientific literature, Pb and Cd concentrations in bone samples obtained from smokers vary and could be placed in the following decreasing order: 10 > 8 > 4.26 > 2.32 > 2.11 > 1.38 > 0.93 > 0.586 and 0.89 > 0.65 > 0.57 > 0.4 > 0.07 > 0.06 > 0.05 > 0.028 mg/kg dm, respectively (see [8, 14, 24, 36–39], this study). While a number of publications indicate a positive correlation between smoking and the incidence of vertebral, forearm, and thigh fractures, we detected a correlation between the concentration of Pb and/or Cd/Ca and the number of cigarettes smoked. Furthermore, a comparison of the concentration ratios in bone samples obtained from the smokers and nonsmokers allowed an assessment of Cd and Pb stress. Brodziak-Dopierała et al. [37] report increased Pb/Fe and Pb/Mn ratios in the bones of male smokers compared with nonsmokers. In this study, we found significantly higher Se/Pb, Se/Cd, Se/THg, and Cd/Ca ratios in the nonsmokers. Most likely, this is a result of Se interacting with the highly toxic elements and displacing them from the metallothionein complex, which leads to a reduction in the toxicity of i.a. Cd, Pb, and THg. Moreover, a correlation analyzed in this study showed an inverse correlation between Se/Pb ratio and the number of cigarettes smoked.

According to the American Dental Association, dental amalgam fillings do not cause any harmful effects; hypersensitivity to THg may occur in approximately 1% of the population. However, people with amalgam fillings do have significantly elevated THg levels in blood, about 3 to 5 times more THg in urine and 2 to 12 times more THg in their body tissues than individuals without dental amalgams. Hajduga and Jędrzejczyk [40] indicate that silver amalgam fillings cause an increase in the levels of THg and Pb in tooth tissues. Available scientific literature finds no relevant comparative data on dental amalgam fillings and THg concentration in bone. In this study, the THg levels in the patients with DA were over 1.5 times higher than in the patients from the NDA group.

Food is considered to be one of the main routes of entry for THg and Se into the human body. Marine fish at the top of the aquatic food chain contain, for instance, elevated levels of both toxic and essential elements, including THg and Se [41]. In Poland, the average Hg contamination of fish and seafood is 0.035 and 0.022 mg/kg, respectively. The consumption of THg in fish and seafood by an adult weighing 60 kg is estimated at 3.2% and 5.6% of PTWI (Provisional Tolerable Weekly Intake). The daily intake of Se in Poland is 0.06 mg, whereas the world average ranges from 0.03 to 0.5 mg—with seafood being one of the major sources of Se [15]. Lanocha et al. [24] show that THg concentrations in cartilage with adjacent compact bone were the lowest in the groups of patients who did not or only rarely ate fish and seafood and were almost twice as high in those consuming fish and seafood several times a month. In this study, there were statistically confirmed differences in Se, THg levels and Se/THg ratio in the tibial plateau between individuals consuming fish and seafood and those who did not prefer such a diet. The lowest concentrations of Se and THg were reported in the NFD group, being higher in FD patients. The tibial bone samples of patients from the FD group were characterized by a smaller Se/THg ratio compared to NFD, although dietary Se is postulated to protect against THg. In our research we observed a negative relationship between Se/THg ratio and fish and seafood diet.

Dietary supplements contain mostly vitamins and minerals and also amino acids, fatty acids, fiber, and various vegetable products, among others. Patients with OA and joint injuries most often supplement themselves with glucosamine, chondroitin, and methylsulfonylmethane, although available research does not unequivocally confirm that the use of such supplements has a positive effect [42]. In this study we observed significant differences in the concentration of Se in the patients receiving dietary supplementation, although it should be pointed out that it may be toxic at higher concentrations, as the difference between the therapeutic and toxic levels is narrow.

Osteolysis surrounding an implant is a complex biochemical process closely linked to the mechanical functioning of the implant. The presence of material implanted in the body and its corrosion leading to metallosis may also become a source of microelements including Fe and cause adverse reactions in the body. In this study, Fe levels in the patients with surgical implants were over two times higher than in the patients without implants. Scientific literature does not provide any appropriate comparative data, although Sidun and Dabrowski [43] have proven the transition of metal ions from the surface of the implant to the bone.

The Osteoarthritis Research Society International recommends only moderate levels of exercise for people with OA to prevent joint overload caused by higher levels of physical activity [44]. In this research we detected a statistically significant negative correlation between Se concentration and sport activity in the patients with OA.

Imbalances in the metabolism of trace metals may lead to metal interactions with potential pathophysiological significance. Knowledge of these correlations is essential for the understanding of kinetic interactions of trace metals in the body [45]. The results of such interactions are highly variable and range from antagonistic to synergistic depending on the metal, its external concentration and exposure, exposure period, and specific organs [14, 17]. We observed positive correlations between THg-Pb and THg-Pb/Cd (Table 5). Additionally, research by Brodziak-Dopierala et al. [14] performed on trabecular bone samples taken from human femoral heads reveals interactions between Pb and Cd with a high correlation coefficient. Bogunia et al. [38] show the correlation coefficient between K-Cd in bone samples. Kwapuliński et al. [32] report that THg is correlated with Pb in cancellous and cortical bone, and we observed a similar relationship in our research. Furthermore, we found antagonistic relationship between element levels and concentration ratios: K-Se/Cd.

Selenium protects against the toxicity of several metals including THg, Pb, and Cd, and in this study we observed negative interactions for the relationship Se-Pb/Cd, Se-Pb/Cd, THg-Se/Pb, THg-Se/THg, Pb-Se/Pb, Pb-Se/Cd, Pb-Se/THg, Cd-Se/Pb, and Cd-Se/Cd, which may neutralize heavy metal toxicity by the formation of poorly soluble selenides excluding those metals from biochemical processes [15]. In addition, selenoproteins are antioxidant enzymes that participate in maintaining cell redox balance, which is important in the regulation of inflammation and bone cell proliferation/differentiation.

## 5. Conclusions

The results reported here may provide a basis for establishing reference values for the tibial plateau in patients with OA who had undergone knee replacement surgery. Our results may lead to a better understanding of the pathogenesis of bone with OA and an improvement in diagnostic efficiency, particularly in patients with symptomatic knee osteoarthritis who smoke, as smoking is a potentially modifiable risk factor.

## Figures and Tables

**Table 1:** Effect of gender on mean values of chemical contents in human tibial plateau from patients after knee surgery (mg/kg dm).

	K	Zn	Mn	Fe	Sr	Se	THg	Pb	Cd	F^−^
Female (*n* = 22)										
AM ± SD	446.80 ± 138.97	98.79 ± 14.15	0.30 ± 0.21	55.98 ± 39.44	45.30 ± 18.59	0.04 ± 0.01	0.005 ± 0.007	1.50 ± 0.69	0.04 ± 0.03	634.54 ± 379.85
Med.	405.22	96.15	0.23	35.17	44.63	0.04	0.0018	1.53	0.03	513.16
Range	295.69–881.57	76.73–129.09	0.08–0.81	12.73–142.97	25.15–116.82	0.02–0.06	0.0007–0.03	0.35–2.91	0.01–0.13	238.40–1815.18
Q_L_–Q_U_	344.48–505.98	89.21–103.70	0.15–0.37	28.46–69.95	34.08–48. 89	0.03–0.05	0.001–0.006	0.96–1.94	0.02–0.05	330.08–893.84

Male (*n* = 11)										
AM ± SD	514.20 ± 157.67	99.12 ± 8.33	0.34 ± 0.30	62.14 ± 38.97	41.6 ± 9.90	0.04 ± 0.02	0.0043 ± 0.003	2.06 ± 1.60	0.06 ± 0.06	569.55 ± 300.49
Med.	455.25	100.55	0.27	49.62	38.65	0.03	0.004	2.06	20.04	449.31
Range	355.04–831.41	89.36–110.82	0.11–0.99	22.51–142.14	34.10–69.73	0.02–0.09	0.001–0.01	1.59–7.28	0.01–0.18	229.30–1153.61
Q_L_–Q_U_	418.73–557.02	89.63–107.9	0.13–0.35	47.41–81.02	34.99–42.88	0.03–0.04	0.002–0.006	1.94–2.60	0.03–0.06	330.96–716.14

Total *n* = 33										
AM ± SD	469.90 ± 146.80	98.90 ± 12.37	0.31 ± 0.24	58.03 ± 38.78	44.10 ± 16.10	0.04 ± 0.03	0.005 ± 0.005	1.87 ± 1.18	0.05 ± 0.04	612.87 ± 351.95
Med.	449.20	97.62	0.23	45.75	41.01	0.04	0.003	1.72	0.04	511.46
Range	295.70–881.60	76.73–129.09	0.08–0.99	12.73–142.97	25.15–116.82	0.02–0.09	0.001–0.03	0.35–7.28	0.01–0.18	229.9–1815.18
Q_L_–Q_U_	370.70–513.34	89.63–103.70	0.15–0.36	28.98–69.95	34.99–46.70	0.03–0.04	0.001–0.006	1.34–2.14	0.02–0.05	330.96–874.01

Female versus Male										
*U*	NS	NS	NS	NS	NS	NS	NS	48	NS	NS
*p*								0.01		

AM: arithmetic mean; SD: standard deviation; Q_L_: lower quartile; Q_U_: upper quartile; *U*: Mann-Whitney *U* test; *p*: level of significance; NS: nonsignificant difference.

**Table 2:** The concentration ratios chemical elements in human tibial plateau from patients after knee surgery (mg/kg dm).

	Se/Pb	Se/Cd	Se/THg	F^−^/Ca · 1000
Female (*n* = 22)				
AM ± SD	0.03 ± 0.02	1.53 ± 1.12	19.13 ± 12.84	41.28 ± 21.81
Med.	0.03	1.15	14.56	40.16
Range	0.009–0.10	0.35–4.93	0.35–4.93	13.19–91.27
Q_L_–Q_U_	0.02–0.03	0.77–2.17	8.56–32.99	22.90–49.70

Male (*n* = 11)				
AM ± SD	0.02 ± 0.02	1.30 ± 1.32	14.74 ± 13.84	39.52 ± 18.74
Med.	0.02	0.94	8.63	34.40
Range	0.01–0.04	0.18–4.5	3.12–47.41	16.40–73.82
Q_L_–Q_U_	0.01–0.02	0.42–1.32	4.70–18.51	19.90–54.97

Total *n* = 33				
AM ± SD	0.03 ± 0.02	1.45 ± 1.17	17.66 ± 13.13	40.69 ± 20.56
Med.	0.02	1.05	13.40	37.71
Range	0.02–0.10	0.18–4.93	0.94–47.41	13.20–91.27
Q_L_–Q_U_	0.02–0.03	0.76–2.13	8.04–31.41	22.90–53.60

Female versus Male				
*U*	35	NS	NS	NS
*p*	0.001			

AM: arithmetic mean; SD: standard deviation; Q_L_: lower quartile; Q_U_: upper quartile; *U*: Mann-Whitney *U* test; *p*: level of significance; NS: nonsignificant difference; F^−^/Ca · 1000: F^−^ concentration in the inorganic portion of bone.

**Table 3:** The concentrations of chemical elements and Se/elements ratios (mg/kg dm) in smokers and nonsmokers among examined patients (mg/kg dm).

Parameter	Mn	Pb	Cd	THg	Se/Pb	Se/Cd	Se/THg
Smokers (*n* = 18)							
AM ± SD	0.31 ± 0.25	2.32 ± 1.38	0.06 ± 0.05	0.01 ± 0.01	0.02 ± 0.02	1.12 ± 1.11	14.18 ± 18.90
Med	0.22	2.09	0.04	0.004	0.02	0.80	8.36
Range	0.08–0.99	0.45–7.28	0.01–0.18	0.001–0.030	0.06–0.08	0.18–4.93	0.94–33.73
Q_L_–Q_U_	0.13–0.36	1.72–2.50	0.03–0.08	0.001–0.006	0.01–0.02	0.44–1.20	6.36–26.61

Nonsmokers (*n* = 15)							
AM ± SD	0.32 ± 0.22	1.34 ± 0.57	0.03 ± 0.03	0.003 ± 0.002	0.04 ± 0.02	1.85 ± 1.16	21.85 ± 13.74
Med	0.28	1.45	0.03	0.002	0.03	1.40	18.52
Range	0.11–0.85	0.35–2.33	0.01–0.13	0.001–0.009	0.02–0.10	0.35–4.49	3.12–47.40
Q_L_–Q_U_	0.15–0.40	0.90–1.67	0.02–0.04	0.001–0.005	0.02–0.05	0.93–2.90	10.17–35.90

Smokers versus Nonsmokers							
*U*	NS	49.0	74	NS	54	72	79
*p*		0.001	0.03		0.01	0.02	0.04

AM: arithmetic mean; SD: standard deviation; Q_L_: lower quartile; Q_U_: upper quartile; *U*: Mann-Whitney *U* test; *p*: level of significance; NS: nonsignificant difference.

**Table 4:** Spearman correlation coefficients between chemical elements content, the concentration ratios, and environmental exposure factors.

	Fe	THg	Pb	Cd	Se/Pb	Se/THg	Cd/Ca	Se	F^−^/Ca · 1000
Age	0.39^xx^	NS	NS	NS	NS	NS	NS	NS	0.30^x^
Implants	0.48^xxx^	NS	NS	NS	NS	NS	NS	NS	NS
Number of cigarettes smoked/day	NS	0.37^xx^	0.71^xxxx^	0.51^xxx^	−0.48^xxx^	NS	0.53^xxx^	NS	NS
Fish and sea food diet	NS	0.42	NS	NS	NS	−0.36^xxxx^	NS	0.32^x^	NS
Sport activity	NS	NS	NS	NS	NS	NS	NS	−0.33^x^	NS
Dental amalgams fillings	NS	NS	NS	NS	NS	NS	NS	NS	NS

Significant level: ^x^ *p* = 0.06; ^xx^ *p* < 0.05; ^xxx^ *p* < 0.01; ^xxxx^ *p* < 0.001; NS: nonsignificant difference; F^−^/Ca · 1000: F^−^ concentration in the inorganic portion of bone.

**Table 5:** Spearman correlation coefficients between chemical elements contents and the concentration ratios in tibial plateau from patients who had undergone knee joint arthroplasty.

	K	Zn	Sr	F^−^	Se	THg	Pb	Cd	Se/Pb	Se/Cd	Se/THg	Cd/Pb	Cd/Ca	F^−^/Ca · 1000
K	—													
Zn	NS	—												
Sr	NS	0.43^xxx^	—											
F^−^	NS	0.36^xx^	NS	—										
Se	NS	NS	NS	NS	—									
THg	NS	NS	NS	NS	NS	—								
Pb	NS	NS	NS	NS	NS	0.53^xx^	—							
Cd	0.42^xx^	NS	NS	NS	NS	NS	0.47^xxx^	—						
Se/Pb	NS	NS	NS	NS	0.47^xxx^	−0.31^xx^	−0.74^xxx^	−0.38^xx^	—					
Se/Cd	−0.41^xx^	NS	NS	NS	0.34^xxx^	NS	−0.32^xx^	−0.90^xxx^	0.46^xxx^	—				
Se/THg	NS	NS	NS	NS	NS	−0.37^xx^	−0.48^xx^	NS	NS	NS	—			
Pb/Cd	NS	NS	NS	NS	−0.38^xx^	NS	NS	NS	NS	NS	NS	—		
Cd/Ca	NS	−0.32^xx^	NS	NS	NS	NS	0.45^xxx^	0.97^xxxx^	NS	−0.88^xxxx^	NS	NS	—	
F^−^/Ca · 1000	NS	NS	NS	0.96^xxxx^	NS	NS	NS	NS	NS	NS	NS	NS	NS	—

Significant level: ^xx^ *p* < 0.05; ^xxx^ *p* < 0.01; ^xxxx^ *p* < 0.001; NS: nonsignificant difference; F^−^/Ca · 1000: F^−^ concentration in the inorganic portion of bone.
